# Superior mesenteric artery syndrome after ileal pouch-anal anastomosis for colon cancer associated with ulcerative colitis: report of a case

**DOI:** 10.1186/s40792-015-0031-7

**Published:** 2015-03-10

**Authors:** Hiroaki Kitade, Takashi Matsuura, Hidesuke Yanagida, Masanori Yamada, Koji Nakai, Katsuji Tokuhara, Takeshi Hijikawa, Kazuhiko Yoshioka, A-Hon Kwon

**Affiliations:** Department of Surgery, Kansai Medical University Takii Hospital, 10-15 Fumizono-cho, Moriguchi, Osaka 570-8507 Japan; Department of Surgery, Kansai Medical University, 2-5-1 Shin-machi, Hirakata, Osaka 573-1191 Japan

**Keywords:** Superior mesenteric artery syndrome, Colorectal cancer, Ileal pouch-anal anastomosis, Pancreatic fistula, Duodenojejunostomy

## Abstract

Superior mesenteric artery syndrome (SMAS) after a surgical operation is very rare. We experienced an extremely rare case of ileal pouch-anal anastomosis with subsequent development of SMAS requiring duodenojejunostomy. A 74-year-old Asian woman underwent total colectomy, ileal pouch-anal anastomosis (J-pouch), covering ileostomy, splenectomy, and distal pancreatectomy for treatment of descending colon cancer associated with ulcerative colitis. She complained of abdominal discomfort and vomiting 17 days postoperatively. Computed tomography (CT) revealed fluid collection at the pancreatic stump. We diagnosed a pancreatic fistula and performed CT-guided drainage. SMAS was thereafter diagnosed by contrast-enhanced CT, which revealed a narrow aortomesenteric angle of 36° and short aortomesenteric distance of 2 mm. The SMAS did not respond to conservative therapy. Finally, we performed duodenojejunostomy. This case illustrates that ileal pouch-anal anastomosis might induce relative stretching of the superior mesenteric artery and flatten it against the aorta, resulting in SMAS.

## Background

Superior mesenteric artery syndrome (SMAS) is a rare disease defined as obstruction of the third portion of the duodenum caused by compression of this region between the superior mesenteric artery (SMA) and aorta. This syndrome was first observed more than 150 years ago. Diagnosis of SMAS is very difficult because of its nonspecific symptoms, including nausea, vomiting, abdominal pain, anorexia, and early satiety. The causes of SMAS are loss of intraabdominal fat, changes in spine extension, and surgical operations [1].

Among patients with SMAS who have a history of surgery (although few), the most common surgical operations are those performed to treat colorectal cancer. In the most recent few decades, ileal pouch-anal anastomosis (IPAA) has been the gold standard reconstruction technique following the performance of total proctocolectomy. IPAA is performed to preserve the sphincter in patients with ulcerative colitis (UC) after total proctocolectomy. The SMA is the main vascular pedicle for the constructed ileal pouch during the performance of IPAA. Stretching of the SMA associated with IPAA might cause SMAS.

To the best of our knowledge, SMAS after IPAA has been reported only five times [2-6]. We herein describe a very rare case of SMAS that developed after IPAA in a patient with descending colon cancer associated with UC.

## Case presentation

A 74-year-old Asian woman with chronic UC was diagnosed with descending colon cancer. She was 144.3 cm tall and weighed 50 kg (body mass index, 24.0 kg/m^2^). A computed tomography (CT) scan revealed invasion of the colon cancer to the tail of the pancreas. Clinical biochemistry and hematology tests showed evidence of slight inflammation. Her carcinoembryonic antigen and carbohydrate antigen levels were within normal limits.

**Figure 1:**
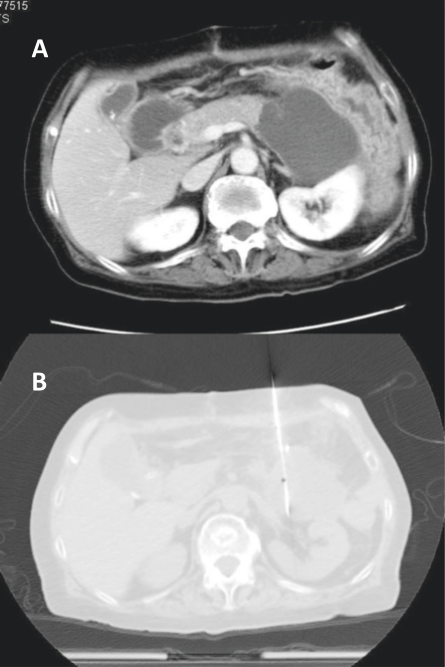
**CT on postoperative day 17. (A)** CT scan revealed fluid collection at stump of the remnant pancreas. **(B)** CT-guided drainage was performed. The amylase level of the fluid was 59,676 U/L.

The patient underwent total colectomy (IPAA) with distal pancreatectomy to treat the pancreatic cancer invasion. On postoperative day 17, she complained of abdominal discomfort and vomiting. A CT scan revealed fluid collection at the pancreatic stump (Figure 1A). We diagnosed a pancreatic fistula (PF) after distal pancreatectomy and performed CT-guided drainage (Figure 1B). The amylase level of the fluid was very high (59,676 U/L).

**Figure 2:**
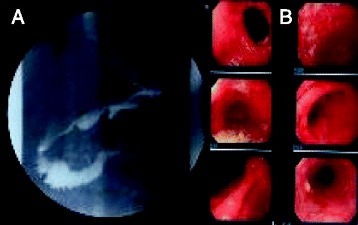
**Upper gastrointestinal series and gastroduodenal endoscopy. (A)** Upper gastrointestinal series revealed an obstruction in the third portion of the duodenum. **(B)** Gastroduodenal endoscopy showed food residue and GERD.

**Figure 3:**
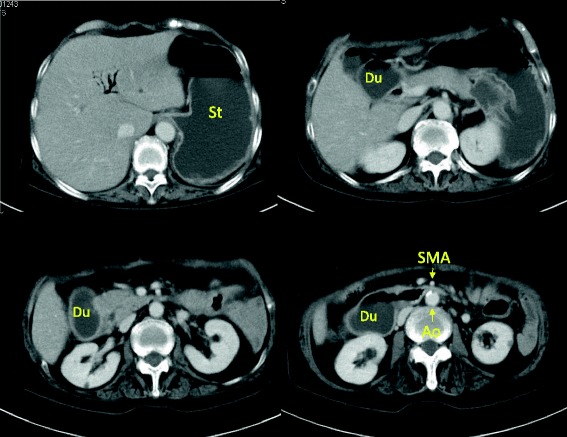
**CT on postoperative day 97.** CT scan demonstrated gastric and duodenal distension and a transition point in the third portion of the duodenum near the takeoff of the SMA. Du, duodenum; St, stomach; Ao, aorta; SMA, superior mesenteric artery.

**Figure 4:**
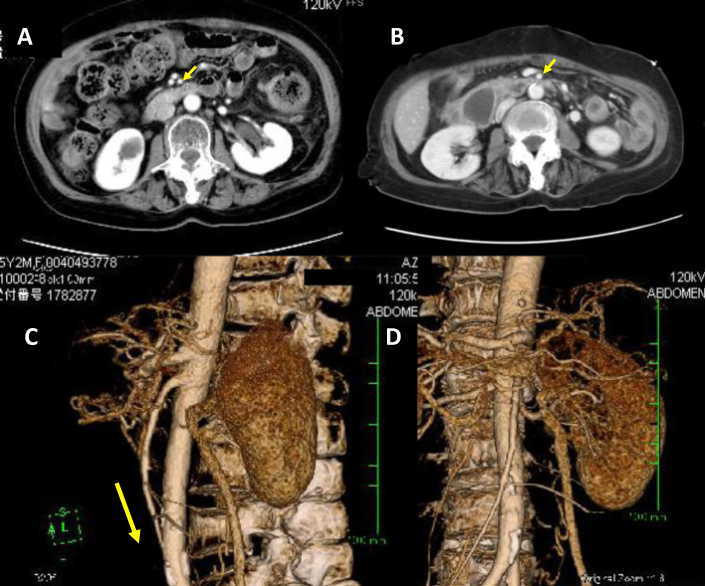
**CT shows a narrow angle and reduced distance between the aorta and SMA. (A)** The distance between the aorta and SMA was 12 mm on preoperative CT. **(B)** The distance between the aorta and SMA on postoperative CTwas 2mm. **(C**, **D)** The angle between the aorta and SMA on postoperative CT was 36°. Localization of the pouch deep in the pelvis causes relative stretching of the SMA and flattens it against the aorta in the retroperitoneum.

Conservative therapy for the PF, including total parenteral nutrition and administration of a proton pump inhibitor, protease inhibitor, and other medications, was not effective. An upper gastrointestinal series revealed an obstruction in the third portion of the duodenum (Figure 2A). Gastroduodenal endoscopy showed food residue and gastroesophageal reflux disease (GERD) (Figure 2B). A CT scan demonstrated gastric and duodenal distension with a transition point in the third portion of the duodenum near the origin of the SMA (Figure 3). In such cases, localization of the pouch deep in the pelvis induces relative stretching of the SMA and flattens it against the aorta within the retroperitoneum. The angle and distance between the aorta and SMA were reduced by the stretched SMA (aortomesenteric angle, 36°; aortomesenteric distance, 2 mm) (Figure 4). We diagnosed this duodenal obstruction as SMAS.

**Figure 5:**
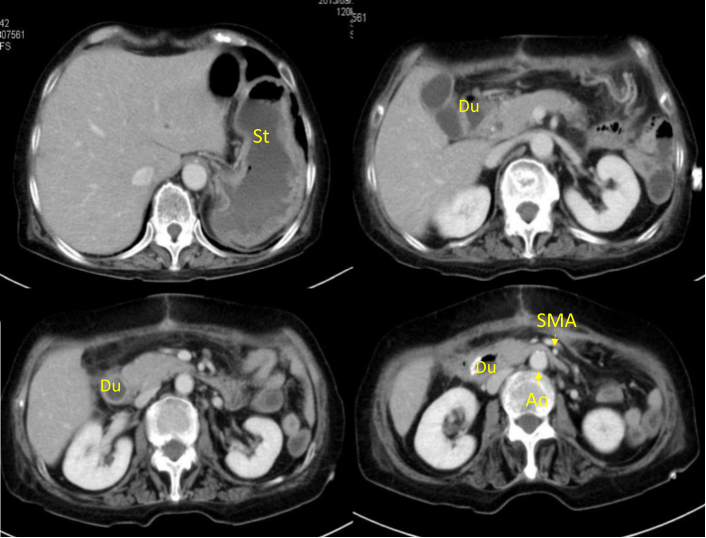
**CT after duodenojejunostomy.** Fluid collection at the pancreatic stump disappeared early after duodenojejunostomy. Du, duodenum; St, stomach; Ao, aorta; SMA, superior mesenteric artery.

Conservative treatment for SMAS and specific treatment for the PF were performed, including administration of an elemental diet, gastrointestinal prokinetic agents, and other medications. However, the patient repeatedly vomited, and the PF did not improve. Finally, side-to-side duodenojejunostomy was performed 110 days postoperatively. After duodenojejunostomy, an upper gastrointestinal series showed no duodenal distension. Gastroduodenal endoscopy showed no signs of GERD or food residue in the stomach. The PF improved soon after the duodenojejunostomy (Figure 5). We removed the drainage tube at the pancreatic stump 13 days postoperatively. The patient was discharged 38 days after the second operation.

## Conclusions

SMAS was first described in 1861 by Rokitansky [7]. In patients with SMAS, the third portion of the duodenum is compressed between the SMA and aorta. The symptoms of SMAS are nonspecific and identical to those of ileus. SMAS is diagnosed by contrast-enhanced CT. The angle between the SMA and aorta (aortomesenteric angle) is normally 45° to 60° [8], and the distance between the SMA and aorta (aortomesenteric distance) usually ranges from 10 to 34 mm [9]. In the present case, the aortomesenteric angle was 36° and the aortomesenteric distance was reduced from 12 mm on preoperative CT to only 2 mm on postoperative CT (Figure 4A, B). An upper gastrointestinal series revealed an obstruction in the third portion of the duodenum (Figure 2A), and a CT scan demonstrated gastric and duodenal distension with a transition point in the third portion of the duodenum near the origin of the SMA (Figure 3). We confirmed that the cause of the duodenal obstruction was SMAS.

**Figure 6:**
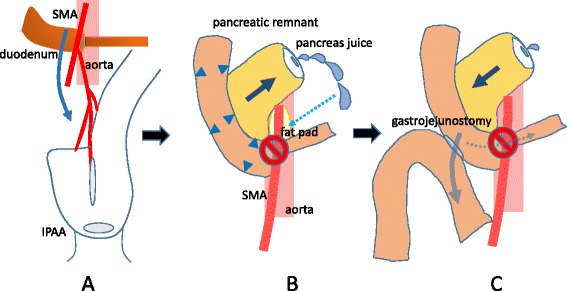
**Schema of the patient’s condition. (A)** Stretching of and traction on the SMA by IPAA caused compression of the third portion of the duodenum (SMAS). **(B)** High pressure in the duodenum caused high pressure at the main pancreatic duct, resulting in PF formation. Pancreatic juice might have reduced the fat pad between the SMA and aorta, worsening SMAS. **(C)** After duodenojejunostomy, the duodenal pressure decreased and the overflow of pancreas juice from the pancreatic stump was subsequently reduced.

One cause of SMAS is believed to be loss of the intraabdominal fat that separates the SMA from the aorta. Other reported causes of SMAS include traction of the SMA and changes in spine extension [1]. There are three populations of patients with SMAS: those who have undergone surgery, those who have experienced severe weight loss, and those with hyperextension of the spine. Among those who have undergone surgery, although few, the most common surgical operations are those performed for treatment of colorectal cancer. In the most recent few decades, IPAA has been the gold standard construction technique following the performance of total proctocolectomy. IPAA is performed for sphincter preservation after total proctocolectomy in patients with UC. Because the SMA is the main vascular pedicle for the constructed ileal pouch during the performance of IPAA, and because the SMA is stretched to some extent, it seems that many cases of SMAS after IPAA should have been reported to date. However, to the best of our knowledge, only five cases of SMAS after IPAA have been reported [2-6]. We suppose that the PF might have affected the development of SMAS in this case. The PF might have developed after SMAS because the drainage volume was small and we were able to remove the drainage tube early after the operation. Furthermore, the fluid collection near the PF was slightly displaced from the obstruction in the duodenum when we performed the drainage. We speculated that the high intraduodenal pressure secondary to SMAS could have created the high pressure at the main pancreatic duct, resulting in prolongation of the PF (Figure 6). Conservative treatment for SMAS reportedly involves an increase in both weight and the mesenteric fat pad [10]; therefore, we initially treated our patient conservatively. However, the patient’s postsurgical weight loss was >10 kg at 3 months postoperatively. The primary cause of SMAS in this case might have been the stretching and traction of the SMA due to the IPAA, which was localized deep in the pelvic cavity. Goes et al. [5] reported that some technical details may make it difficult for the pouch to reach the anal canal. Therefore, undesirable tension could be transmitted to the root of the mesentery, narrowing the aortomesenteric angle and increasing the risk of duodenal compression. In addition to the stretching of and traction on the SMA, the presence of a PF might affect the reduction of the fat pad between the SMA and aorta because pancreatic lipase digests fat. Appropriate nutritional management normally increases the fat pad between the SMA and aorta, but the vicious circle between the PF and SMAS might interfere with this process. Truong et al. [2] recently reported a case of SMAS after IPAA; conservative treatment was effective, and the symptoms disappeared 4 weeks later.

Surgical treatments that may be considered for SMAS include laparoscopic, robotic, or open duodenojejunostomy [10-13]. In the present case, we chose open duodenojejunostomy because of the history of open total colectomy and drainage of the PF. The patient’s symptoms improved, and gastroduodenal endoscopy showed no GERD or food residue after this operation. The fluid collection at the pancreatic stump disappeared soon after the duodenojejunostomy (Figure 5), indicating that the cause of the PF was SMAS.

In summary, we experienced a very rare case of SMAS after construction of an IPAA. The SMAS was induced by stretching of and traction on the SMA associated with IPAA. The SMAS caused high pressure to develop in the duodenum and main pancreatic duct, resulting in PF formation. This PF might have reduced the fat pad between the SMA and aorta, resulting in prolonged SMAS. Surgical treatment was finally required (Figure 6). Management of SMAS is normally conservative. However, inappropriate nutritional management and other complications might affect the treatment of SMAS. We should keep in mind that SMAS can occur after IPAA.

## Consent

Written informed consent was obtained from the patient for publication of this case report and accompanying images. A copy of the written consent is available for review by the Editor-in-Chief of this journal.

## Abbreviations

CT: computed tomography
GERD: gastroesophageal reflux disease
IPAA: ileal pouch-anal anastomosis
PF: pancreatic fistula
UC: ulcerative colitis
SMA: superior mesenteric artery
SMAS: superior mesenteric artery syndrome
